# Are Peripartum Changes in CCL2 Associated with Maternal Metabolic Status?

**DOI:** 10.3390/cimb48020143

**Published:** 2026-01-28

**Authors:** Aleksandra Obuchowska-Standyło, Żaneta Kimber-Trojnar, Katarzyna Trojnar, Monika Czuba, Bożena Leszczyńska-Gorzelak

**Affiliations:** 1 Chair and Department of Obstetrics and Perinatology, Medical University of Lublin, 20-090 Lublin, Poland; bozena.leszczynska-gorzelak@umlub.edu.pl; 2 Student’s Scientific Association at the Chair and Department of Obstetrics and Perinatology, Medical Faculty, Medical University of Lublin, 20-090 Lublin, Poland; katarzynaatrojnar@gmail.com; 3 Department of Clinical Genetics, Medical University of Lublin, 20-080 Lublin, Poland; monika.czuba@umlub.edu.pl

**Keywords:** CCL2, monocyte chemoattractant protein-1, bioelectrical impedance analysis, gestational weight gain, maternal weight change, pregnancy, postpartum

## Abstract

C-C motif chemokine ligand 2 (CCL2) may reflect subtle metabolic–inflammatory changes in pregnancy. This study evaluated CCL2 concentrations and their peripartum changes in women with uncomplicated term pregnancies, focusing on associations with maternal metabolic status. Serum CCL2 was measured before delivery and 48 h postpartum; urinary CCL2 was assessed postpartum. Peripartum serum change (ΔCCL2) was calculated. BMI was recorded pre-pregnancy (or early pregnancy), at delivery, and 48 h postpartum; total BMI change (ΔBMI) was derived. Participants were stratified into two groups (ΔBMI > 1 kg/m^2^ vs. ≤1 kg/m^2^). Peripartum serum CCL2 changes differed significantly between ΔBMI groups. In the total cohort, CCL2 correlated with HbA1c and selected body composition indices, including fat tissue index, lean tissue index, and body cell mass. In women with ΔBMI > 1 kg/m^2^, additional associations were found with BMI, peripartum BMI change, HbA1c, ferritin, creatinine, and total body water. Among women with ΔBMI ≤ 1 kg/m^2^, significant relationships were observed with uric acid and triglycerides. Peripartum CCL2 dynamics appear to reflect maternal metabolic status, even in metabolically “normal” pregnancies, but these findings are exploratory and should be interpreted cautiously. CCL2 is a promising marker of subtle metabolic alterations in late pregnancy and the early postpartum period, but further validation is required before clinical application.

## 1. Introduction

C-C motif chemokine ligand 2 (CCL2), also known as monocyte chemoattractant protein-1 (MCP-1), is a key mediator of immune–metabolic interactions and has been implicated in the pathophysiology of obesity, insulin resistance (IR), and type 2 diabetes (T2DM) [1,2,3]. Dysregulation of the CCL2–CCR2 axis contributes to chronic low-grade inflammation and has been linked to metabolic and inflammatory disorders, including gestational diabetes mellitus (GDM) [3].

C-C motif chemokine ligand 2 (CCL2), also known as monocyte chemoattractant protein-1 (MCP-1), is an important mediator of immune–metabolic interactions and has been implicated in the pathophysiology of obesity, IR, and T2DM [1,2,3]. Dysregulation of the CCL2–CCR2 axis contributes to chronic low-grade inflammation and has been linked to metabolic and inflammatory disorders, including obesity-related inflammation and GDM [3].

During pregnancy, CCL2 is expressed at the maternal–fetal interface—including trophoblasts, decidual tissue, and the myometrium—and maternal obesity has been associated with activation of the CCL2/CCR2 pathway [4,5]. These mechanisms may influence maternal metabolic adaptations during late gestation; however, the temporal patterns of CCL2 secretion around delivery remain poorly characterized. Moreover, the early postpartum period is marked by rapid changes in body weight, fluid balance, and body composition, but the relationship between these physiological shifts and cytokine dynamics has not been sufficiently explored. Understanding this interplay may help generate hypotheses regarding the links between metabolic stress, subclinical inflammation, and peripartum immunological responses.

Therefore, the aim of this exploratory study was to evaluate peripartum CCL2 concentrations in women with uncomplicated term pregnancies and to examine their associations with selected maternal metabolic and anthropometric parameters.

## 2. Materials and Methods

Among 100 initially recruited women, 56 were excluded due to chronic diseases or pregnancy-related complications. The final cohort consisted of 44 Caucasian women with uncomplicated singleton term pregnancies who delivered at the Chair and Department of Obstetrics and Perinatology in Lublin. Eligible participants met the following criteria: age 20–43 years, singleton pregnancy, and term delivery (37–40 weeks of gestation). Women with overweight or obesity were included provided they had no history of metabolic or cardiovascular disease and no pregnancy complications.

Exclusion criteria included multiple pregnancy, history of endocrine disorders, GDM, diabetes mellitus, hypertension, preeclampsia (PE), thyroid disorders, epilepsy, malignancies, presence of pacemaker, use of medications affecting metabolic parameters, and contraindications to bioelectrical impedance analysis (BIA). Information regarding medical history and exclusion criteria was verified through both patient interviews and review of medical records.

Anthropometric measurements and BMI calculation:

BMI was recorded at three time points: before or in early pregnancy (based on pregnancy card and physician’s entry), on the day of delivery (prior to labor), and 48 h postpartum. From these measurements, three BMI change indices were calculated:

- ΔBMI_gestational (ΔBMI_g): Difference between BMI on the day of delivery and pre-pregnancy BMI;
- ΔBMI_puerperal (ΔBMI_p): Difference between BMI on the day of delivery and BMI 48 h postpartum;
- ΔBMI (total BMI change): Difference between BMI 48 h postpartum and pre-pregnancy BMI.

Participants were stratified into two groups based on ΔBMI: the group with ΔBMI > 1 kg/m^2^ (*n* = 22), which included women with greater overall BMI change, and the group with ΔBMI ≤ 1 kg/m^2^ (*n* = 22), which comprised women with minimal or negative BMI change. The threshold of ΔBMI > 1 kg/m^2^ was selected because this degree of change is considered clinically meaningful in maternal weight dynamics and may be associated with differences in metabolic and obstetric outcomes; it was also guided by the exploratory distribution of our dataset.

Biochemical analysis:

Venous blood samples were collected before delivery and 48 h postpartum, after a 6 h fasting period. First-morning urine samples were collected 48 h postpartum. Maternal serum and urine concentrations of CCL2 were measured using the Human CCL2/MCP-1 ELISA Kit–Quantikine (DCP00, R&D Systems, Minneapolis, MN, USA) according to the manufacturer’s instructions. Serum CCL2 was measured at two time points: before delivery and 48 h postpartum; urinary CCL2 was measured on the second postpartum day.

Assay details: Solid phase sandwich ELISA, 96-well plate, assay length 3.5–4.5 h, sample volume 100 μL (serum/urine), sensitivity 10 pg/mL, assay range 31.2–2000 pg/mL, natural and recombinant human MCP-1 specificity, <0.5% cross-reactivity with related molecules, no significant interference observed. ELISA was selected because chemokine concentrations vary between assay types; this method provides sensitive, direct protein quantification, and target proteins are more stable than their mRNA transcripts [6].

Body composition analysis:

Maternal body composition and hydration status were evaluated at 48 ± 2 h postpartum using BIA with the Body Composition Monitor (BCM; Fresenius Medical Care, Bad Homburg, Germany). Measurements were performed in the supine position under standardized conditions. Routine postpartum care ensured adequate hydration and early mobilization, which were consistent across all participants. BIA-derived parameters included total body water (TBW), extracellular-to-intracellular water ratio (ECW/ICW), lean tissue index (LTI), fat tissue index (FTI), and body cell mass (BCM). Body composition was assessed only postpartum, as antenatal BIA measurements are limited by physiological changes in pregnancy.

Ethical approval:

The study was approved by the Bioethics Committee of the Medical University of Lublin (approval no. KE-0254/61/2020), and written informed consent was obtained from all participants in accordance with ethical standards for research involving human subjects.

Statistical analysis:

Statistical analyses were performed using IBM SPSS Statistics, version 26 (IBM Corp., Armonk, NY, USA). Quantitative data were presented as mean, median, standard deviation (SD), minimum and maximum, and first and third quartiles. Normality was assessed using the Shapiro–Wilk test. Differences between two groups were evaluated using the Mann–Whitney U test, and correlations were assessed using Spearman’s rank correlation coefficient. Multivariable regression analyses were not performed due to the limited sample size and the risk of model overfitting.

All analyses were exploratory and hypothesis-generating. Tests were two-tailed, and a *p*-value < 0.05 was considered statistically significant. No formal correction for multiple testing was applied due to the exploratory nature of the analyses and the limited sample size; therefore, the risk of type I error should be considered when interpreting the results.

## 3. Results

There were no statistically significant differences in absolute BMI values between the two groups at any of the three assessment points—pre-pregnancy, on the day of delivery, or 48 h postpartum (Table 1).

The distribution of participants across pre-pregnancy BMI categories (underweight/normal weight, overweight, and obese) was also similar between the groups. In contrast, all three BMI change indices—ΔBMI_g, ΔBMI_p, and ΔBMI—differed significantly between the groups (*p* < 0.001 for all comparisons). Specifically, ΔBMI_g was higher in the ΔBMI > 1 kg/m^2^ group, reflecting greater weight gain during pregnancy, while ΔBMI_p indicated a larger early postpartum BMI reduction in the ΔBMI ≤ 1 kg/m^2^ group. Consequently, these differences were consistent with the predefined group stratification criteria. No statistically significant differences were observed between groups for body composition parameters measured by BIA (total body water (TBW), extracellular to intracellular water (ECW/ICW) ratio, lean tissue index (LTI), fat tissue index (FTI), body cell mass (BCM)), or for biochemical markers including glucose, insulin, HOMA-IR, HbA1c, lipid profile, uric acid, ferritin, or homocysteine.

In the overall study population, serum CCL2 concentrations were 170.47 ± 86.07 pg/mL (median—158.06) before delivery and 139.99 ± 68.76 pg/mL (median—124.62) at 48 h postpartum. Urinary CCL2 measured at 48 h postpartum was 643.71 ± 748.34 pg/mL (median—361.88).

The change in serum CCL2 (ΔCCL2) was calculated as the difference between the 48 h postpartum and pre-delivery concentrations. The mean ΔCCL2 was −2.52 ± 48.41 pg/mL (median—0.64) in the ΔBMI ≤ 1 kg/m^2^ group and −58.45 ± 78.70 pg/mL (median—47.79) in the ΔBMI > 1 kg/m^2^ group. Detailed results are presented in Table 2.

No significant differences were observed between the two groups in serum CCL2 before or after delivery, nor in urinary CCL2. However, a statistically significant difference was found for ΔCCL2 (*p* = 0.01), with a larger decrease in serum CCL2 observed in women with greater total BMI increase (ΔBMI > 1 kg/m^2^), whereas women with minimal BMI change (ΔBMI ≤ 1 kg/m^2^) showed almost no change.

Given the exploratory nature of the study and the limited sample size, correlation analyses were performed in an unadjusted manner. Subsequently, correlations between serum and urinary CCL2 concentrations, as well as ΔCCL2, and BMI indices were analyzed in the overall study population and within each ΔBMI subgroup. In the entire cohort, a statistically significant negative correlation was observed between ΔCCL2 and ΔBMI. In the ΔBMI ≤ 1 kg/m^2^ group, no statistically significant correlations were found between CCL2 concentrations and any BMI indices. In contrast, in the ΔBMI > 1 kg/m^2^ group, predelivery serum CCL2 correlated positively with BMI on the day of delivery (*p* = 0.042) and BMI at 48 h postpartum (*p* = 0.040). Additionally, a statistically significant positive correlation was observed between urinary CCL2 in the early puerperium and ΔBMI_p (*p* = 0.031). Detailed results are presented in Table 3.

In the overall study population, predelivery serum CCL2 concentrations correlated positively and significantly with HbA1c (*p* = 0.026) and FTI (*p* = 0.036). Positive correlations were also observed between urinary CCL2 and LTI (*p* = 0.016) as well as BCM (*p* = 0.037). Additionally, ΔCCL2 showed a statistically significant negative correlation with HbA1c (*p* = 0.043), showing an inverse association between HbA1c and ΔCCL2. No significant correlations were found between CCL2 concentrations and the remaining laboratory parameters or body composition measures. Detailed results are presented in Table 4.

Restricting the analysis to women in the ΔBMI ≤ 1 kg/m^2^ group, a statistically significant positive correlation was observed between postpartum serum CCL2 concentration and uric acid (*p* = 0.043). In addition, ΔCCL2 showed a statistically significant negative correlation with triglyceride levels (*p* = 0.028). No other significant correlations were found between CCL2 concentrations and laboratory parameters or body composition measures in this subgroup. Detailed results are presented in Table 5.

In the ΔBMI > 1 kg/m^2^ group, a statistically significant negative correlation was observed between pre-delivery serum CCL2 and creatinine (*p* = 0.026), as well as a positive correlation between post-delivery serum CCL2 and TBW (*p* = 0.026). A statistically significant negative correlation was also found between urinary CCL2 and ferritin (*p* = 0.014). Additionally, a significant negative correlation was observed between ΔCCL2 and HbA1c (*p* = 0.007), demonstrating an inverse association between HbA1c and ΔCCL2 in this subgroup. Details are provided in Table 6.

## 4. Discussion

The current literature lacks evidence regarding how gestational weight gain—not only defined as excessive gestational weight gain (EGWG) but also considered as suboptimal adipose tissue gain leading to metabolic changes—affects maternal metabolic and immunological adaptations. In particular, relatively little attention has been paid to heterogeneous or subclinical patterns of adipose tissue accumulation that may still influence inflammatory pathways. Because pregnancy physiologically increases adipose tissue mass in women, it is also associated with activation of inflammatory pathways [7,8]. Therefore, in the natural history of pregnancy, both the placenta and adipose tissue may contribute to inflammation during pregnancy, as reflected by elevated levels of circulating inflammatory markers [9]. In women with excessive adipose tissue accumulation during pregnancy, subclinical inflammation appears to be exacerbated as physiological changes (e.g., placental secretion) coincide with the pathological expansion of hormonally active adipose tissue. This, in turn, may increase the risk of pregnancy-related metabolic complications, such as GDM. In metabolic diseases, CCL2 appears to contribute to carbohydrate metabolism disorders, including IR and diabetes (and its complications, including diabetic nephropathy) [10].

In this study, we analyzed changes in BMI expressed in kg/m^2^ rather than conventional gestational weight gain in kilograms. Common criteria consider adequate or excessive weight gain stratified by pre-pregnancy BMI, but kilograms alone do not account for maternal height, which has clinical significance. BMI, by normalizing weight for height, provides a more objective measure and allows comparisons between women of different statures. Significant differences were observed between the ΔBMI ≤ 1 kg/m^2^ and ΔBMI > 1 kg/m^2^ groups in three indices: BMI increase during pregnancy (ΔBMI_g), BMI decrease in the early postpartum period (ΔBMI_p), and total BMI change from pre-pregnancy to 48 h postpartum (ΔBMI). These indices comprehensively reflect maternal weight fluctuations, with ΔBMI particularly valuable as it captures total BMI change while minimizing confounding from fetal growth, amniotic fluid, placental weight, maternal blood volume, and fluid retention. Pre-pregnancy BMI was similar in both groups, indicating differences arose from weight change patterns rather than baseline weight.

ΔBMI_g accounts for pregnancy-related factors such as fetal weight, amniotic fluid, placental weight, maternal fluid expansion, fat accumulation, and uterine/breast enlargement; it differed significantly between groups, reflecting distinct patterns of gestational weight change. The population included only uncomplicated singleton term pregnancies; cases with oligohydramnios or polyhydramnios were excluded. Neonatal birth weight and postpartum maternal body composition did not differ between groups. Interestingly, although the ΔBMI ≤ 1 kg/m^2^ group showed a smaller increase in gestational BMI (*p* < 0.001), they experienced a significantly greater decline in BMI in the early postpartum period compared to the study group (*p* < 0.001). This suggests different patterns of maternal weight change between groups both during pregnancy and immediately after delivery.

The choice of ΔBMI > 1 kg/m^2^ as a stratification threshold is supported by clinical evidence indicating that even relatively small changes in BMI have measurable metabolic consequences. A gain of ≥1 kg/m^2^ has been associated with increased risk of GDM, gestational hypertension, preeclampsia, cesarean delivery, and large for gestational age (LGA) infants, with risk escalating further for gains of 1–3 kg/m^2^ and more than doubling for gains ≥ 3 kg/m^2^ [11,12,13,14,15,16,17]. Conversely, a BMI decrease ≥1 kg/m^2^ in women with overweight or obesity may reduce the risk of GDM and LGA, though it can increase the risk of SGA infants [15,16,17]. Similarly, in non-pregnant individuals with diabetes or prediabetes, even a 1 kg/m^2^ change in BMI is associated with changes in HbA1c, blood pressure, and lipid profile, with the largest effect observed in T2DM, moderate in prediabetes, and smallest in normoglycemic individuals [18]. Therefore, using ΔBMI > 1 kg/m^2^ is clinically meaningful, reflecting a threshold beyond which metabolic and inflammatory changes may become pronounced, justifying its use for stratification in this study.

In the group with a BMI > 1 kg/m^2^, a significantly greater decrease in CCL2 levels was observed from pre-delivery to early postpartum (*p* = 0.01). Given the lack of differences in CCL2 levels measured before and after delivery, this result suggests that the groups differ primarily in the dynamics of peripartum change rather than in values at single time points. This observation may reflect differences in inflammatory regulation associated with distinct maternal weight trajectories. Furthermore, a statistically significant inverse correlation between ΔCCL2 and ΔBMI was observed in the entire cohort, suggesting that changes in CCL2 levels are directly related to the reduction in BMI across the peripartum period.

In the group with ΔBMI > 1 kg/m^2^, there was a statistically significant positive correlation between serum CCL2 concentration before delivery and BMI on the day of delivery (*p* = 0.042) and BMI on the second day of the postpartum period (*p* = 0.04). Furthermore, a statistically significant positive correlation was observed between urinary CCL2 concentration in the early postpartum period and ΔBMI_p (*p* = 0.031). The interpretation of urinary CCL2 remains complex, potentially reflecting renal clearance, local inflammation, or both; this highlights the need for further studies assessing urinary chemokine dynamics longitudinally. However, in the group with ΔBMI ≤ 1 kg/m^2^, no correlation with BMI was observed.

In a study by Friis et al., assessing the dynamics of CCL2 concentrations during pregnancy depending on pre-pregnancy BMI, CCL2 levels differed significantly between BMI categories in early pregnancy; however, in late pregnancy, these differences between categories were no longer significant, despite higher baseline CCL2 concentrations in women who were overweight or obese before conception [9]. However, among Danish pregnant women, higher pre-pregnancy BMI was associated with elevated CCL2 levels in the third trimester of pregnancy [19]. Another study conducted in pregnant women without GDM at 28 weeks of gestation showed that CCL2 levels increased with increasing BMI [20]. A meta-analysis showed that CCL2 levels are higher in women with GDM than in healthy pregnant women [1]. CCL2 levels were examined in a mouse model of GDM, revealing high CCL2 expression in visceral adipose tissue and placental tissue; subsequent administration of an anti-CCL2 antibody (αCCL2) alleviated GDM symptoms and reduced inflammation [3].

In our entire sample, pre-partum CCL2 levels correlated positively and significantly with HbA1c (*p* = 0.026), indicating that higher HbA1c levels were associated with higher CCL2 concentrations. Furthermore, a statistically significant linear negative correlation was observed between ΔCCL2 and HbA1c (*p* = 0.043), similarly observed in the ΔBMI > 1 kg/m^2^ group (*p* = 0.007), suggesting that higher HbA1c levels are associated with a greater decline in CCL2 during labor. No comparable correlations were found in the ΔBMI ≤ 1 kg/m^2^ group. No significant correlations were found for fasting glucose, insulin, or HOMA-IR. Given that elevated CCL2 levels have been reported in women with GDM [1], these findings suggest that subtle metabolic differences, even within normoglycemic ranges, may be reflected in inflammatory dynamics.

It is worth noting that both groups were comparable in terms of body composition and routine laboratory parameters in the early postpartum period, including HbA1c levels. In the group of women with a ΔBMI ≤ 1 kg/m^2^, a positive correlation was observed between CCL2 concentration and serum uric acid concentration (*p* = 0.043) after delivery, and a negative correlation was observed between delta CCL2 and triglyceride concentration (*p* = 0.028). Similar correlations were not observed in the group with a ΔBMI > 1 kg/m^2^. These associations may indicate distinct metabolic–inflammatory coupling mechanisms in women with minimal total BMI change and should be interpreted as hypothesis-generating.

We found no studies in the available literature examining the correlation between CCL2 concentration and body composition measurements assessed by BIA in the early postpartum period. Therefore, our findings are particularly noteworthy. Considering the entire cohort of women, prepartum serum CCL2 levels were found to correlate positively with FTI (*p* = 0.036). A positive correlation was also observed between postpartum urinary CCL2 levels and LTI (*p* = 0.016) and BCM (*p* = 0.037). In the group with ΔBMI > 1 kg/m^2^, a positive correlation was also observed between postpartum CCL2 levels and TBW (*p* = 0.026). In light of these findings, future studies should consider additional modifiers such as breastfeeding, peripartum blood loss, and mode of delivery It appears that overnutrition, along with imbalances between fetal and maternal immunosuppression and tolerance, may contribute to persistent low-grade inflammation in pregnancy—a feature often observed in preeclampsia, preterm birth, and GDM [21,22,23].

### Limitations

The main limitation of this study is the relatively small sample size. Recruitment was challenging due to strict inclusion criteria, including only healthy pregnant women without chronic illnesses, pregnancy complications, or multiple pregnancies. This limited the availability of participants with overweight or obesity, making a larger, representative sample difficult. The increasing prevalence of overweight and obesity among pregnant women is observed not only in Poland but worldwide. Although women with higher BMI were included to reflect the distribution typical of the Polish obstetric population, generalizability to the broader population, especially those with preexisting metabolic disorders or non-Caucasian populations, may be limited. Additionally, maternal body composition was assessed only in the early postpartum period, and CCL2 dynamics were evaluated at only two time points (pre-delivery and 48 h postpartum), which may not capture important changes during labor, delivery, and the early postpartum period. Intermediate or later postpartum measurements could alter interpretation. Analyses were unadjusted for potential confounders such as maternal age, pre-pregnancy BMI, and mode of delivery, which may influence CCL2 dynamics and body composition.

Given the exploratory nature of multiple correlation analyses, results should be interpreted with caution as no correction for multiple testing was applied. These findings are hypothesis-generating and not yet suitable for clinical stratification.

Future studies should include larger, more diverse populations, assess additional time points for CCL2, and consider potential modifiers such as breastfeeding, peripartum blood loss, labor duration, and lifestyle factors.

## 5. Conclusions

Our findings suggest that CCL2 dynamics during the perinatal period may reflect maternal metabolic status, even in the absence of overt disturbances in glucose metabolism. This may indicate that CCL2 could be sensitive to subtle metabolic and inflammatory changes in late pregnancy and early postpartum, potentially complementing standard glycemic markers. Given the limited sample size and exploratory nature of this study, these observations should be interpreted with caution. Further research in larger, more diverse, and high-risk populations is needed to determine clinical relevance, reproducibility, and potential confounding effects.

If confirmed, assessment of CCL2—particularly in women with greater BMI increases—could provide additional insights into perinatal metabolic adaptation. Currently, CCL2 should be regarded as a promising but still investigational marker requiring additional validation before any clinical application.

## Figures and Tables

**Table 1:** Pre-pregnancy BMI categories, BMI values at key time points, BMI change indices, body composition, and biochemical parameters in ΔBMI groups.

Variable	ΔBMI > 1 kg/m^2^ (*n* = 22)	ΔBMI ≤ 1 kg/m^2^ (*n* = 22)	*p*
Pre-pregnancy BMI (kg/m^2^)	27.05 ± 7.21	30.04 ± 8.08	0.204
**Pre-pregnancy BMI categories (*n*)**			
Underweight + normal weight (<25)	10	6	0.658
Overweight (25–29.9)	8	6	0.477
Obese (≥30)	4	10	0.776
BMI on day of delivery (kg/m^2^)	32.12 ± 7.62	32.54 ± 7.51	0.854
BMI 48 h postpartum (kg/m^2^)	30.07 ± 7.56	28.86 ± 8.01	0.610
Gestational BMI change ΔBMI_g (kg/m^2^)	5.06 ± 2.15	2.50 ± 1.01	<0.001 *
Puerperal BMI change ΔBMI_p (kg/m^2^)	2.05 ± 0.98	3.68 ± 1.51	<0.001 *
Total BMI change ΔBMI (kg/m^2^)	3.01 ± 2.16	−1.18 ± 1.42	<0.001 *
Total body water (%)	35.79 ± 6.69	34.87 ± 6.71	0.503
Extracellular water to intracellular water index	0.93 ± 0.13	0.92 ± 0.18	0.353
Lean tissue index (kg/m^2^)	12.63 ± 1.83	13.34 ± 3.36	0.597
Fat tissue index (kg/m^2^)	17.05 ± 7.64	15.29 ± 8.41	0.313
Body cell mass (kg)	19.13 ± 4.53	19.76 ± 6.93	0.925
Glucose (mg/dL)	79.77 ± 8.19	81.36 ± 8.03	0.510
Insulin (mIU/L)	11.28 ± 4.28	11.66 ± 5.11	0.869
HOMA-IR	2.27 ± 1.01	2.38 ± 1.21	0.681
HbA1c (%)	5.35 ± 0.33	5.29 ± 0.39	0.860
High-density lipoprotein (mg/dL)	77.64 ± 20.67	72.14 ± 16.82	0.467
Low-density lipoprotein (mg/dL)	133.45 ± 29.49	142.86 ± 27.54	0.318
Triglycerides (mg/dL)	173.82 ± 30.57	182.27 ± 26.96	0.391
Uric acid (mg/dL)	5.12 ± 0.78	5.05 ± 0.88	0.897
Ferritin (µg/L)	13.36 ± 7.16	16.18 ± 10.78	0.510
Homocysteine (µmol/L)	10.75 ± 3.05	10.71 ± 2.56	0.981

Values are mean ± SD unless otherwise indicated; *p*-values calculated using Mann–Whitney U test; * statistically significant (*p* < 0.05); ΔBMI_g—gestational BMI change (delivery BMI − pre-pregnancy BMI); ΔBMI_p—puerperal BMI change (delivery BMI—48 h postpartum BMI); ΔBMI—total BMI change (48 h postpartum BMI—pre-pregnancy BMI); HOMA-IR—insulin resistance index.

**Table 2:** Serum and urinary CCL2 concentrations before and after delivery and their changes by ΔBMI group (pg/mL).

Parameter	ΔBMI ≤ 1 kg/m^2^ (Mean ± SD, Median)	ΔBMI > 1 kg/m^2^ (Mean ± SD, Median)	*p*
CCL2 in serum before delivery	150.98 ± 71.68, 145.74	189.96 ± 96.10, 159.93	0.162
CCL2 in serum at 48 h postpartum	148.46 ± 73.60, 128.80	131.51 ± 64.14, 111.08	0.496
ΔCCL2 (48 h postpartum − pre-delivery)	−2.52 ± 48.41, −0.64	−58.45 ± 78.70, −47.79	0.01 *
CCL2 in urine at 48 h postpartum	606.11 ± 735.34, 275.52	681.31 ± 776.54, 399.60	0.526

Values are mean ± SD unless otherwise indicated; *p*-values calculated using Mann–Whitney U test; * statistically significant (*p* < 0.05); ΔBMI—total BMI change (48 h postpartum BMI − pre-pregnancy BMI).

**Table 3:** Correlations between CCL2 concentrations and BMI indices in total population and by ΔBMI groups.

Group/CCL2	Pre-Pregnancy BMI	BMI on Delivery Day	BMI 48 h Postpartum	ΔBMI_g (Gestational)	ΔBMI_p (Puerperal)	ΔBMI (Total)
**Total population**						
serum before delivery	r = 0.212 *p* = 0.168	r = 0.278 *p* = 0.068	r = 0.292 *p* = 0.055	r = 0.198 *p* = 0.198	r = −0.069 *p* = 0.655	r = 0.219 *p* = 0.154
serum after delivery	r = 0.143 *p* = 0.353	r = 0.127 *p* = 0.412	r = 0.064 *p* = 0.682	r = −0.014 *p* = 0.926	r = 0.145 *p* = 0.348	r = −0.105 *p* = 0.497
ΔCCL2	r = −0.096 *p* = 0.535	r = −0.185 *p* = 0.229	r = −0.258 *p* = 0.091	r = −0.262 *p* = 0.086	r = 0.150 *p* = 0.331	**r = −0.340** *p* = 0.024 *
urine after delivery	r = −0.017 *p* = 0.910	r = 0.018 *p* = 0.909	r = −0.001 *p* = 0.996	r = −0.042 *p* = 0.784	r = 0.178 *p* = 0.249	r = −0.068 *p* = 0.660
**ΔBMI ≤ 1 kg/m^2^ group**						
serum before delivery	r = 0.152 *p* = 0.500	r = 0.111 *p* = 0.622	r = 0.150 *p* = 0.505	r = −0.179 *p* = 0.425	r = −0.071 *p* = 0.755	r = −0.016 *p* = 0.942
serum after delivery	r = −0.084 *p* = 0.710	r = −0.108 *p* = 0.631	r = −0.083 *p* = 0.713	r = 0.016 *p* = 0.942	r = −0.093 *p* = 0.681	r = 0.051 *p* = 0.820
ΔCCL2	r = −0.263 *p* = 0.238	r = −0.256 *p* = 0.251	r = −0.260 *p* = 0.242	r = 0.141 *p* = 0.533	r = −0.121 *p* = 0.590	r = 0.091 *p* = 0.687
urine after delivery	r = 0.061 *p* = 0.787	r = 0.071 *p* = 0.755	r = 0.023 *p* = 0.919	r = −0.266 *p* = 0.231	r = 0.069 *p* = 0.760	r = −0.206 *p* = 0.359
**ΔBMI > 1 kg/m^2^ group**						
serum before delivery	r = 0.367 *p* = 0.093	**r = 0.436** *p* = 0.042 *	**r = 0.441** *p* = 0.040 *	r = 0.315 *p* = 0.153	r = −0.268 *p* = 0.228	r = 0.150 *p* = 0.504
serum after delivery	r = 0.265 *p* = 0.232	r = 0.300 *p* = 0.175	r = 0.224 *p* = 0.317	r = 0.181 *p* = 0.420	r = 0.373 *p* = 0.088	r = −0.099 *p* = 0.660
ΔCCL2	r = −0.239 *p* = 0.284	r = −0.299 *p* = 0.176	r = −0.325 *p* = 0.139	r = −0.272 *p* = 0.221	r = −0.143 *p* = 0.526	r = −0.173 *p* = 0.440
urine after delivery	r = −0.009 *p* = 0.968	r = 0.050 *p* = 0.826	r = −0.015 *p* = 0.946	r = −0.059 *p* = 0.795	**r = 0.460** *p* = 0.031 *	r = −0.371 *p* = 0.090

r—Spearman’s correlation coefficient; *—statistically significant (*p* < 0.05); ΔCCL2—difference between CCL2 concentration in serum after and before delivery; ΔBMI_g—gestational BMI change (delivery day–pre-pregnancy BMI); ΔBMI_p—puerperal BMI change (delivery day–48 h postpartum BMI); ΔBMI—total BMI change (48 h postpartum–pre-pregnancy BMI).

**Table 4:** Correlations between CCL2 and laboratory parameters and body composition measures in the total study population.

Parameter	Total Study Population (*n* = 44)			
	CCL2 in Serum Before Delivery (pg/mL)	CCL2 in Serum After Delivery (pg/mL)	ΔCCL2 (pg/mL)	CCL2 in Urine After Delivery (pg/mL)
HbA1c (%)	r = 0.336, *p* = 0.026 *	r = 0.086, *p* = 0.578	r = −0.306, *p* = 0.043 *	r = 0.112, *p* = 0.469
Glucose (mg/dL)	r = 0.059, *p* = 0.705	r = −0.079, *p* = 0.609	r = −0.023, *p* = 0.880	r = −0.143, *p* = 0.353
Insulin (mIU/L)	r = 0.038, *p* = 0.808	r = 0.075, *p* = 0.630	r = 0.050, *p* = 0.748	r = −0.184, *p* = 0.233
HOMA-IR	r = 0.025, *p* = 0.871	r = 0.033, *p* = 0.830	r = 0.070, *p* = 0.652	r = −0.216, *p* = 0.158
HDL (mg/dL)	r = −0.014, *p* = 0.926	r = −0.076, *p* = 0.626	r = −0.035, *p* = 0.821	r = 0.041, *p* = 0.793
LDL (mg/dL)	r = −0.13, *p* = 0.402	r = 0.024, *p* = 0.878	r = 0.109, *p* = 0.483	r = −0.009, *p* = 0.955
Triglycerides (mg/dL)	r = 0.094, *p* = 0.545	r = −0.141, *p* = 0.361	r = −0.270, *p* = 0.076	r = −0.039, *p* = 0.800
Uric acid (mg/dL)	r = 0.217, *p* = 0.158	r = 0.108, *p* = 0.483	r = −0.072, *p* = 0.644	r = 0.034, *p* = 0.828
Creatinine (mg/dL)	r = −0.165, *p* = 0.283	r = −0.113, *p* = 0.463	r = 0.155, *p* = 0.314	r = −0.080, *p* = 0.604
Ferritin (µg/L)	r = −0.023, *p* = 0.881	r = 0.006, *p* = 0.967	r = 0.075, *p* = 0.628	r = −0.248, *p* = 0.104
Homocysteine (µmol/L)	r = 0.249, *p* = 0.103	r = 0.256, *p* = 0.093	r = −0.050, *p* = 0.749	r = 0.134, *p* = 0.387
TBW (%)	r = 0.216, *p* = 0.159	r = 0.223, *p* = 0.145	r = −0.080, *p* = 0.605	r = 0.263, *p* = 0.084
ECW/ICW	r = 0.142, *p* = 0.357	r = 0.166, *p* = 0.283	r = −0.020, *p* = 0.896	r = −0.173, *p* = 0.261
LTI (kg/m^2^)	r = −0.021, *p* = 0.893	r = 0.021, *p* = 0.892	r = −0.015, *p* = 0.922	r = 0.360, *p* = 0.016 *
FTI (kg/m^2^)	r = 0.317, *p* = 0.036 *	r = 0.005, *p* = 0.975	r = −0.285, *p* = 0.060	r = −0.205, *p* = 0.182
BCM (kg)	r = 0.019, *p* = 0.904	r = 0.082, *p* = 0.599	r = 0.008, *p* = 0.960	r = 0.315, *p* = 0.037 *

*p*—Mann–Whitney U test; *—difference statistically significant (*p* < 0.05); r—Spearman’s correlation coefficient; ΔCCL2—difference between CCL2 concentration in serum after and before delivery; HOMA-IR—insulin resistance index; HDL—high-density lipoprotein; LDL—low-density lipoprotein; TBW—total body water; ECW/ICW—extracellular water to intracellular water index; LTI—lean tissue index; FTI—fat tissue index; BCM—body cell mass.

**Table 5:** Correlations between CCL2 and laboratory parameters and body composition measures in the Δ BMI ≤ 1 kg/m^2^ group.

Parameter	Δ BMI ≤ 1 kg/m^2^ Group			
	CCL2 in Serum Before Delivery (pg/mL)	CCL2 in Serum After Delivery (pg/mL)	ΔCCL2 (pg/mL)	CCL2 in Urine After Delivery (pg/mL)
HbA1c (%)	r = 0.297, *p* = 0.180	r = 0.290, *p* = 0.191	r = −0.187, *p* = 0.404	r = 0.221, *p* = 0.324
Glucose (mg/dL)	r = 0.209, *p* = 0.351	r = 0.080, *p* = 0.724	r = −0.113, *p* = 0.616	r = 0.091, *p* = 0.686
Insulin (mIU/L)	r = 0.252, *p* = 0.257	r = 0.152, *p* = 0.500	r = −0.075, *p* = 0.740	r = −0.253, *p* = 0.256
HOMA-IR	r = 0.205, *p* = 0.360	r = 0.127, *p* = 0.573	r = −0.034, *p* = 0.879	r = −0.210, *p* = 0.349
HDL (mg/dL)	r = −0.127, *p* = 0.575	r = 0.042, *p* = 0.851	r = 0.221, *p* = 0.322	r = −0.064, *p* = 0.776
LDL (mg/dL)	r = −0.380, *p* = 0.081	r = −0.129, *p* = 0.568	r = 0.146, *p* = 0.517	r = −0.137, *p* = 0.542
Triglycerides (mg/dL)	r = 0.058, *p* = 0.799	r = −0.143, *p* = 0.526	r = −0.469, *p* = 0.028 *	r = 0.184, *p* = 0.411
Uric acid (mg/dL)	r = 0.348, *p* = 0.113	r = 0.435, *p* = 0.043 *	r = 0.199, *p* = 0.374	r = −0.044, *p* = 0.844
Creatinine (mg/dL)	r = 0.106, *p* = 0.639	r = 0.164, *p* = 0.464	r = 0.184, *p* = 0.412	r = 0.005, *p* = 0.983
Ferritin (µg/L)	r = 0.230, *p* = 0.304	r = 0.067, *p* = 0.767	r = −0.099, *p* = 0.661	r = −0.043, *p* = 0.850
Homocysteine (µmol/L)	r = 0.208, *p* = 0.354	r = 0.235, *p* = 0.293	r = −0.075, *p* = 0.436	r = 0.103, *p* = 0.648
TBW (%)	r = 0.067, *p* = 0.766	r = 0.026, *p* = 0.909	r = −0.075, *p* = 0.740	r = 0.229, *p* = 0.306
ECW/ICW	r = 0.218, *p* = 0.330	r = 0.137, *p* = 0.543	r = 0.059, *p* = 0.793	r = −0.133, *p* = 0.555
LTI (kg/m^2^)	r = −0.208, *p* = 0.353	r = −0.108, *p* = 0.633	r = −0.026, *p* = 0.909	r = 0.358, *p* = 0.102
FTI (kg/m^2^)	r = 0.286, *p* = 0.197	r = −0.008, *p* = 0.970	r = −0.280, *p* = 0.207	r = −0.181, *p* = 0.421
BCM (kg)	r = −0.132, *p* = 0.559	r = −0.094, *p* = 0.678	r = −0.084, *p* = 0.711	r = 0.407, *p* = 0.060

*p*—Mann–Whitney U test; *—difference statistically significant (*p* < 0.05); r—Spearman’s correlation coefficient; ΔCCL2—difference between CCL2 concentration in serum after and before delivery; HOMA-IR—insulin resistance index; HDL—high-density lipoprotein; LDL—low-density lipoprotein; TBW—total body water; ECW/ICW—extracellular water to intracellular water index; LTI—lean tissue index; FTI—fat tissue index; BCM—body cell mass.

**Table 6:** Correlations between CCL2 and laboratory parameters and body composition measures in the ΔBMI > 1 kg/m^2^ group.

Parameter	ΔBMI > 1 kg/m^2^ Group			
	CCL2 in Serum Before Delivery (pg/mL)	CCL2 in Serum After Delivery (pg/mL)	ΔCCL2 (pg/mL)	CCL2 in Urine After Delivery (pg/mL)
HbA1c (%)	r = 0.370, *p* = 0.090	r = −0.141, *p* = 0.530	r = −0.561, *p* = 0.007 *	r = −0.081, *p* = 0.721
Glucose (mg/dL)	r = −0.103, *p* = 0.648	r = −0.286, *p* = 0.197	r = 0.003, *p* = 0.990	r = −0.301, *p* = 0.174
Insulin (mIU/L)	r = −0.115, *p* = 0.611	r = −0.022, *p* = 0.922	r = 0.082, *p* = 0.717	r = −0.020, *p* = 0.931
HOMA-IR	r = −0.153, *p* = 0.498	r = −0.132, *p* = 0.559	r = 0.091, *p* = 0.685	r = −0.198, *p* = 0.378
HDL (mg/dL)	r = 0.020, *p* = 0.930	r = −0.123, *p* = 0.585	r = −0.163, *p* = 0.469	r = 0.105, *p* = 0.643
LDL (mg/dL)	r = 0.149, *p* = 0.507	r = 0.155, *p* = 0.490	r = −0.112, *p* = 0.618	r = 0.171, *p* = 0.448
Triglycerides (mg/dL)	r = 0.178, *p* = 0.428	r = −0.160, *p* = 0.476	r = −0.212, *p* = 0.344	r = −0.227, *p* = 0.310
Uric acid (mg/dL)	r = 0.121, *p* = 0.591	r = −0.238, *p* = 0.286	r = −0.360, *p* = 0.100	r = 0.080, *p* = 0.722
Creatinine (mg/dL)	r = −0.474, *p* = 0.026 *	r = −0.363, *p* = 0.097	r = 0.291, *p* = 0.189	r = −0.200, *p* = 0.372
Ferritin (µg/L)	r = −0.287, *p* = 0.195	r = −0.105, *p* = 0.641	r = 0.189, *p* = 0.400	r = −0.515, *p* = 0.014 *
Homocysteine (µmol/L)	r = 0.270, *p* = 0.224	r = 0.274, *p* = 0.217	r = −0.159, *p* = 0.479	r = 0.097, *p* = 0.669
TBW (%)	r = 0.267, *p* = 0.229	r = 0.473, *p* = 0.026 *	r = −0.025, *p* = 0.911	r = 0.213, *p* = 0.341
ECW/ICW	r = 0.034, *p* = 0.881	r = 0.235, *p* = 0.292	r = 0.076, *p* = 0.736	r = −0.219, *p* = 0.329
LTI (kg/m^2^)	r = 0.179, *p* = 0.425	r = 0.235, *p* = 0.292	r = −0.022, *p* = 0.922	r = 0.334, *p* = 0.128
FTI (kg/m^2^)	r = 0.321, *p* = 0.145	r = 0.002, *p* = 0.994	r = −0.317, *p* = 0.151	r = −0.296, *p* = 0.181
BCM (kg)	r = 0.085, *p* = 0.706	r = 0.229, *p* = 0.306	r = 0.034, *p* = 0.881	r = 0.280, *p* = 0.207

*p*—Mann–Whitney U test; *—difference statistically significant (*p* < 0.05); r—Spearman’s correlation coefficient; ΔCCL2—difference between CCL2 concentration in serum after and before delivery; HOMA-IR—insulin resistance index; HDL—high-density lipoprotein; LDL—low-density lipoprotein; TBW—total body water; ECW/ICW—extracellular water to intracellular water index; LTI—lean tissue index; FTI—fat tissue index; BCM—body cell mass.

## Data Availability

The raw data supporting the conclusions of this article will be made available by the authors on request.
